# Shall parent / patient wishes be fulfilled in any case? A series of 32 ethics consultations: from reproductive medicine to neonatology

**DOI:** 10.1186/s12910-018-0342-x

**Published:** 2019-01-08

**Authors:** Mirella Muggli, Christian De Geyter, Stella Reiter-Theil

**Affiliations:** 1 Department of Clinical Ethics, Psychiatric Hospitals of the University Basel, University Hospital, University of Basel, Wilhelm Klein-Strasse 27, CH-4002 Basel, Switzerland; 2 Institute of Reproductive Medicine and Gynaecological Endocrinology (RME), University Hospital, University of Basel, Vogesenstrasse 134, CH-4031 Basel, Switzerland

**Keywords:** Ethics consultation, Reproductive medicine, Obstetrics, Neonatology, Parent/ patients’ wishes, Patient autonomy

## Abstract

**Background:**

Questions concerning the parent/ patient’s autonomy are seen as one of the most important reasons for requesting Ethics Consultations (ECs). Respecting parent/ patient’s autonomy also means respecting the patient’s wishes. But those wishes may be controversial and sometimes even go beyond legal requirements. The objective of this case series of 32 ECs was to illustrate ethically challenging parent / patients’ wishes during the first stages of life and how the principle of patient’s autonomy was handled.

**Methods:**

The case series has a qualitative retrospective approach. A documentary sheet was designed de novo and information was gained from EC minutes and medical charts. The cases originate from the following specialties: reproductive medicine, obstetrics and neonatology as well as two interdisciplinary cases.

**Results:**

Through the structured EC minutes aspects of patient / parents’ wishes could be identified explicitly. Overall the patient / parents’ wishes were **not** supported in **61% of the cases**. Central reasons for rejection of patient / parent wishes were mainly the protection of the best interest of the unborn / new-born child as well as the rejection of clinical approaches that were regarded as being substandard treatment.

**Conclusion:**

The study shows that treatment decisions in reproductive medicine, obstetrics and neonatology raise substantial ethical questions leading to the request for ethics consultation. The systematic case series presented here gives insight into the ethical reflection carried out to support the clinicians in their decision-making and counselling. It shows that clinicians, after using ethics consultation, make deliberate decisions that do not “automatically” fulfil the treatment requests of the patients and parents (to-be).

## Background

In the constantly growing and increasingly complex world of present-day medicine the stage between conception and birth is one of the most vulnerable. It’s often complicated by ethical dilemmas.

The ethical community agrees on the importance of ethics consultations (ECs) in the daily routine of hospitals. Pfäfflin and co-workers [1] state that “clinical ethics consultation (CEC) has gained increasing importance in Europe during the past decade”. Not only do ECs offer support in ethically challenging situations, they have even been shown to be “…associated with reduced consumption of medical resources.” [2] There is still a lack of research on the implementation of EC in the time period between conception and birth.

We first performed a literature search to document this gap. After the literature search, a case series of ECs performed in reproductive medicine, obstetrics and neonatology was undertaken to fill this gap and to give insight into ethical reasoning in a particular area of medicine with unique ethical questions and challenges. During our analytical process it became obvious that most of the ECs were requested because of controversial and sometimes borderline illegal wishes of patients and parents (to-be). Therefore, the focus and research question of this paper is to investigate what ethical reasons underlie the decisions whether to offer the desired treatment, or not.

## Methods

First a systematic literature search was conducted following the PRISMA Statement [3, 4] to study ECs in reproductive medicine, obstetrics and neonatology. Eligible records were identified by searching the following electronic databases: Pubmed, DIMDI, DRZE and Ethxweb using the keywords: ethics consultation, ethical dilemma, ethics case study, moral deliberation, reproductive medicine, obstetrics, pregnancy, abortion, infanticide, sanctity of life, neonatology, preterm infants (keywords were translated for German databases) for records published in English or German. Further eligibility criteria were any common features with the medical field specialties (reproductive medicine, obstetrics and neonatology) of our study in relation to ethical dilemma and clinical EC.

**Fig. 1:**
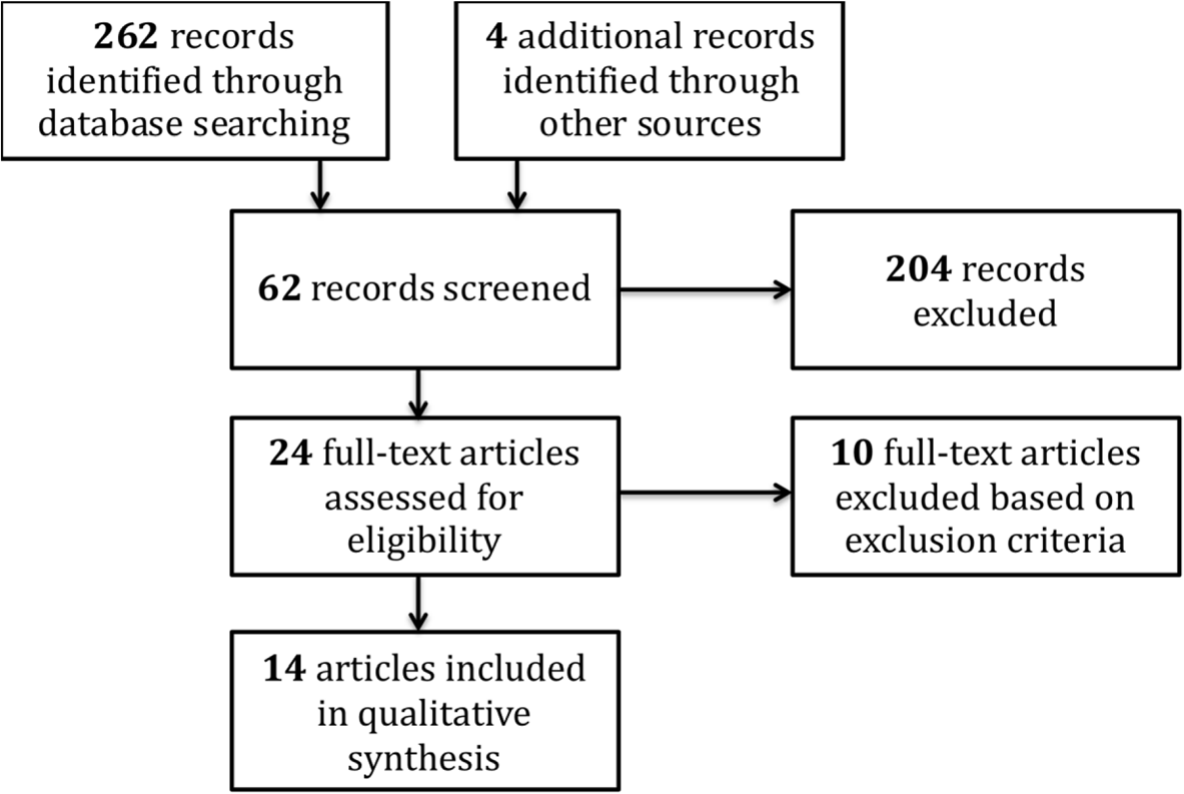
Schematic representation of the literature search work flow

Sixty-two articles were initially selected by title or abstract and were screened again for eligibility. Records that concerned a very local problematic from a single nation differing strongly from Central European or Western context, were not included. Single case reports were also eliminated. This second screening round resulted in 24 records for which a full-text screening for eligibility was carried out leading to the exclusion of another 10 records for various reasons (e.g. “ethics” in the title or abstract with no further mentioning in the article itself) [5] . The full selection process is schematically shown in Fig. 1.

The main result of this literature search was that there were almost no records dealing with the selected topic. The full-text analysis of only 14 eligible records [5–18] revealed a picture of great diversity that is difficult to reduce to a common denominator. Topics range from the conflict between wishes of parents-to-be / patient autonomy on the one hand and a clinician’s intention to provide best medical care on the other, between non-maleficence and beneficence in reproductive medicine [6], over general overview of common ethical problems occurring in the pre-, peri- and postnatal period in obstetrics [8], to the ethical implications of prenatal diagnosis and the termination of the pregnancy by feticide. Because the results of our literature review were sparse and varied widely no meta-analysis was conducted.

Overall only 3 specific studies dealing with EC activity were identified. Tapper et al. analysed all ECs held in a teaching hospital over 3 years. Although this retrospective review is not specific to our field of interest, leaving reproductive medicine or neonatology unmentioned, this paper does hold some interesting findings about ECs in obstetrics. According to their study obstetrics is one of the services that required the most time-intensive consultations: “The ethical issues manifest in pregnancy are famously turbulent, (...)” [11]. Streuli et al. analysed 95 ECs held at the biggest Children’s Hospital in Switzerland and they state that EC “are interventions with possible side effects and should undergo follow-up research (…)”. In their population 17% of the cases were neonates. The most common issue was withdrawal or withholding of a specific treatment (44%). Also the parents’ wishes are well documented (mostly for maximum treatment; in 31% of the cases) [5]. A recent study analysed 100 CECs including a meeting and full documentation held in the somatic (USB) and the psychiatric (UPK) university hospitals, Basel, over 3 years. From the 50 USB-ECs 26% were requested by general gynaecologists. Prenatal and assisted reproduction conflicts (22%) were among the most frequently observed ethical issues [18]. These numbers from different institutions and studies all point toward the need of EC in this particular clinical area. Also, the articles illustrate the diversity of ethical dilemmas, which may evolve out of this sensitive part of medical practice.

A retrospective qualitative analysis of a series of cases was then carried out to identify the kinds of wishes of parents (to-be) or patients that the clinicians regarded as ethically problematic motivating them to request ethics consultation. We used existing EC records [18] complemented by a documentary form adjusted to evaluate ECs in neonatology [19, 20]. According to Mayrings approach for qualitative analysis we created categories that display the wishes of the patients/ parents (to-be) [21]. After this structuring process those categories were applied onto the documentary forms to identify those wishes.

The information acquisition is based on the “Embedded Researcher” approach, in which the ethics researcher is given full access to the hospital ward, the patients’ records as well as the treating physicians and the nursing team allowing him to familiarise himself with the context of each case. Data collection is carried out without disturbing or intervening into the on-going processes in the hospital. This procedure allows for a close look into the heart of the ethical dilemma in daily clinical practice [22]. The structured documentation includes a short summary of the underlying medical problem and a detailed analysis of the EC. The documentation achieves a high level of standardisation; moreover it makes the EC process transparent and comprehensible for any third party [18, 23, 24]. The adjusted form was tested with a selected case from each of the three investigated fields of specialisation to rule out any inadequacies.

Theoretically and methodologically, our EC approach is based on the four-principles approach of biomedical ethics (Beauchamp and Childress) [25] as well as a systematic change of perspectives. By actively acknowledging everyone’s interest we can “ensure fair consideration of the views of all the parties involved” [26]. The goal of this approach is to reduce any bias towards one side. Another central element of our EC approach is a comparison and evaluation of possible different diagnostic and therapeutic options in every case. All options are analysed under the ethical aspects of the four-principle approach [25–27]. The whole procedure allows to take a closer look at the most relevant needs, rights and obligations of all persons involved [28].

A total of **32** EC cases of the Women’s University Hospital of Basel (UFK) and the Children’s University Hospital of Basel (UKBB) were analysed, all of them were held during a period from 2002 until 2016: 8 in reproductive medicine, 15 in obstetrics, 7 in neonatology and 2 interdisciplinary cases. Included were only cases, in which the Department of Clinical Ethics was involved for an EC session and full documentation was available. Not involved, however, were informal discussions dealing with ethical aspects within the team or in educational meetings on ethical issues [28]. The author did not actively participate in the any of the EC sessions. All EC records used for this study were stored in an anonymised version.

ECs in our study are carried out “… on a professional basis in a ‘small team-approach’.” [18] Most ECs were requested by the treating physicians. They were chaired by an experienced clinical ethicist leading the discussion and to assure that the patients/parents’ views were represented explicitly. This aspect is of particular importance when the patients / parents are not directly participating in the discussion, as in most cases. Most meetings were inter-professional including physicians, nurses, midwives, psychologists, social workers together with the clinical ethicists’ staff. The patients / parents are most of the times involved indirectly through information by the physician in charge before the EC and its outcomes afterwards to guarantee shared decision making [18].

## Results

Through the structured EC minutes all aspects of patient / parents’ wishes, ethical reasoning and outcomes were identified explicitly. A short summary of every case studied in this work is given in Table 1.

**Table 1:** Case Chart

Fields	No	Ethical focus of presented problem(s)	Wishes of patient / parents	Wishes supported
Reproductive Medicine	R1	Infertile husband, diagnosed with cystic fibrosis, wishing treatment with assisted reproduction	ICSI despite uncertain life expectancy of the father-to-be	Yes
	R2	Infertile multimorbid husband with severe Diabetes mellitus Type 2	ICSI despite uncertain life expectancy of the father-to-be	Yes
	R3	After termination of pregnancy of healthy twin pregnancy after ICSI using donated semen	Wish for another treatment with ICSI	No
	R4	Infertile husband and failed testicular biopsy	Sperm donation of husband’s father	No by EC changed into Yes by head physician.
	R5	Infertile husband with osteogenesis imperfecta	ICSI despite possible risk of giving on the disease	Yes
	R6	Multimorbid wife with kidney failure and extremely rare blood type	ICSI despite possible deadly complications during pregnancy and delivery	Yes by EC changed into No by head physician.
	R7	Wife with multiple sclerosis and unfulfilled desire to have children	ICSI despite progressive disabling sickness	No
	R8	Wife with several miscarriages after ICSI and borderline personality disorder which worsened after miscarriage causing conflict with the team	Repetition of ICSI	Yes
Obstetrics	S1	Twin pregnancy with impending delivery, need for lung-maturation-treatment after reproductive treatment abroad	Refusal of lung maturation; only “healthy” children to survive	No
	S2	Request for abortion in the 16th week because of gastroschisis	Abortion after 12th week in case of a treatable disease	No
	S3	Triplet pregnancy after ICSI and risk for preeclampsia	Selective feticide	Yes
	S4	PPROM in 21st week, lack of compliance (fundamental evangelical church)	Refusal of treatment insisting on God’s direct support	No
	S5	Healthy twin pregnancy after ICSI and denial of selective feticide	Abortion of both unborn children	No
	S6	Diagnosis of trisomy 18 in 25th week	Comfort care and refusal of life-sustaining measurements	Yes
	S7	Request for termination of pregnancy in the 14th week because of minor hand malformation, no impact on child’s QoL	Termination of pregnancy after 12th week in case of minor malformation	No
	S8	Incompetent patient (low IQ), life-threatening pregnancy	Patient’s father requests abortion	Yes
			Patient’s father requests for sterilisation	No
	S9	Request for termination of pregnancy in the 21st week by the 15yo pregnant woman	Abortion after 12th week	No
	S10	Request for termination of pregnancy in 22nd week because of cleft lip and palate	Abortion after 12th week in case of treatable disease	No
	S11.1	Unborn child with a complex of multiple diseases not compatible with life in the 26th week of pregnancy	Carrying out pregnancy but no medical intervention	Yes
	S11.2	Unborn child with a complex of multiple diseases not compatible with life in the 33rd week of pregnancy	Giving birth to a living child by caesarean section	Yes
	S12	Request for abortion in 22nd week because of distal forearm malformation	Abortion after 12th week in case of treatable disease	No
	S13	Single mother with a strict Muslim background requests termination of pregnancy in the 22nd week	Abortion after 12th week in case of psychosocial distress	Yes
	S14	Premature rupture of membranes in 20th week of pregnancy	Request for determined advice for further procedure	No
Neonatology	N1	Limitation vs. intensification of treatment in preterm new-born with brain haemorrhage	Drug-addicted mother requests maximum treatment	No
	N2	Parents of full term new-born with asphyxia request EC to discuss further treatment limitation	Treatment limitation of life-sustaining measurements incl. Fluid supply	No
	N3	Full term new-born with apallic syndrome after intracranial haemorrhage. Planning of further steps	Father requested treatment limitation incl. food & fluid supply referring to outdated guideline	No
	N4	Full term new-born with VACTERL association. Planning of further steps	Wish for “perfect child”, vote in favour of surgery	No
	N5	Treatment limitation in preterm-born infant with extended brain damage	Limitation of food supply	No
	N6	Preterm new-born with trisomy 18 in mosaic. Planning of further steps	Parents (Muslims) designate the child “a gift from God” and wish maximum treatment	No
	N7	Treatment limitation in case of severe asphyxia + maternal postpartum death	Widowed father initially requested DNR	No
Neurosurgery	NC1	Pregnant women with intracerebral bleeding in 21st week of pregnancy and unborn child with multiple diseases. Termination vs. continuation of pregnancy regarding prognosis of both mother and child	Continuation of pregnancy	Yes
Family Planning	F1	Patient with severe depression with unfulfilled desire to have children after having two abortions because of her mental condition	Wish for removal of IUD	(Yes)

In reproductive medicine the wishes of the parents-to-be were supported in 5 out of 8 cases, while they were not supported in 3 cases. In 2 cases the participating head clinician overruled the EC outcome after changing his mind (from yes to no, or vice versa). The majority of cases (9/15) in obstetrics resulted with a NO regarding support of patient / parent wishes. In neonatology all 7 EC cases were concluded with a NO responding to parent wishes. In 2 interdisciplinary cases from neurosurgery and family planning patients’ / parents’ wishes were supported in both cases. Overall the patient / parents’ wishes were not supported in 61% of all cases.

Central reasons for not supporting and thus not following the preferences of patient / parents were mainly the protection of the best interest of the unborn / new-born child as well as the rejection of a clinical approach that was regarded as substandard treatment [24, 29–33].

### What does “YES” or “NO” mean?

#### Yes

In reproductive medicine a YES means that the couples wish for infertility treatment was supported, sometimes even despite difficulties or doubts relating to existing morbidity in the woman (cases R6; R8) or regarding the acceptability of the preferred sperm donor (R4).

#### No

In some cases of requested abortion in obstetrics, the EC came to the conclusion that abortion should not be offered (6/8 cases). However, patients or couples may have had the abortion carried out elsewhere.

Facing a pregnant patient’s non-adherent behaviour on the basis of fundamentalist religious beliefs, clinical staff supported by EC tried to explicitly persuade the patient to follow the therapeutic recommendation (staying in hospital and undergoing a C-section; S4). In individual cases it meant that the EC outcome encouraged the clinical staff to persuade the (initially reluctant) patient to give the recommended prenatal treatment a try (lung maturation; S1).

## Discussion

In reproductive medicine most of the requests for EC made by clinical staff were discussed with reference to the Swiss law on assisted reproductive medicine (Bundesgesetz über die medizinisch unterstützte Fortpflanzung vom 18. Dezember 1998, (Fortpflanzungsmedizingesetz, FMedG; SR 810.11)). One of the most challenging parts of this law is that “... [assisted reproduction treatment] should only be used, when the future welfare of the child is foreseeable and secured” [34] until it reaches the legal age of 18 years.

Of the three cases in which patient / parents’ wishes were not supported, two were rejected by explicit reference to this paragraph, highlighting the different possible interpretations and disagreement.

In one case (R4) a risk for the future child’s wellbeing was anticipated at a psychosocial level with possible problems resulting from the suggested sperm donor being the patient’s father-in-law, i.e. a situation where father and grandfather would appear in personal union. While in the second case (R7) one of the major concerns was that a progressive debilitating sickness, i.e. multiple sclerosis, could impair normal care of the new-born. Lastly in the third case (R3), to be discussed in detail later, the wish for yet another fertility treatment after repeated abortion following previously administered fertility treatment was denied, mainly to protect the ethical integrity (working ethos) of the team from being instrumentalized.

In obstetrics, as noted before, 2/3 of the mothers-to-be wishes were not supported. This result can be explained to a certain extent by reference to the Swiss penal code regulating legal abortion (Schweizerisches Strafgesetzbuch vom 21. Dezember 1937 (StGB; SR 311.0)). In 5 out of 8 cases the wish for abortion after the 12th week of pregnancy (termination of pregnancy up until week 12 is legal) was denied. In all these cases the underlying medical diagnosis was not regarded sufficient to justify abortion since beyond this limit of 12 weeks, abortion is only allowed if ending the pregnancy can prevent great physical or psychological harm.

It can be stated that especially in those two areas of medicine the law leaves ample room for interpretation. The resulting void may be filled by a clinical ethics consultation. It summarizes and puts together all legal, medical and ethical aspects in order to reach the best possible result and the most comprehensive agreement on the basis of a sound ethical analysis.

Also, in neonatology, ECs may play an important role in advocating in favour of the new-born child serving its best interest. However, the astonishing rate of 100% rejection of parents’ wishes in our case series raises some questions: Do neonatologists request EC only in severe cases with highly controversial parents’ wishes? Does the high rejection rate imply a lack of respect for parental rights? Do the EC-meetings permit deciding overprotectively pro-child?

From a neonatologist’s point of view new-borns are at a disadvantage. Janvier and co-workers stated in 2008: “new-born infants and particularly pre-term infants are systematically devalued, (...)” [35]. The fundamental right for comprehensive medical care is at stake if the patient is a new-born child. It is the legal right of the parents to decide, what’s best for their child. However, they have a moral obligation to act and decide at their child’s best interest, not just following their own preferences. Yet, if they decide in favour of a particular treatment, which may cause lifelong disability for the child, it not only affects the new-born but the entire family as well. Facing such consequences, it can be hard for parents to decide with only the child’s best interest on their minds while disregarding their own. Therefore, the principle of respect for autonomy is not simply enforceable when the patient is a new-born child [29].

This emphasises the importance of ECs. By way of the systematic change of perspectives [23] not only the parents’ wishes, beliefs and needs can be expressed, but also the unspoken interest of the new-born child.

Although, as illustrated before, each of the main subgroups has its unique characteristics, there is a common theme: the appeal of patients/ parents-to-be to medicine as a wish-fulfilling institution. It’s a “relatively new umbrella term denoting any sort of medical treatment carried out without direct medical need.” [36] Other fields in medicine, such as cosmetic surgery, clearly are included in this category. However, there is a big grey area and it is difficult to define the margin between conventional vs. wish-fulfilling medicine. Two main differences are “the process of coming to an indication for employing a medical procedure, and, closely related to this, the process of decision making.” [37] It is an approach to the patient’s autonomy in its most direct form. The patient may ask for a treatment directly, often without the presence of acute symptoms, and skip important steps in the decision process, such as counselling on different options and their pros and cons. Consenting to a treatment (otherwise the end point of a medical appointment) appears to be an already given fact or even the catalyst to seeking medical help.

When applied to ethical questions concerning unborn or new-born children this way of practicing one’s autonomy receives a further dimension: the decisions on treatment options are always made by the parents (to-be) in substitution of the patient (the new-born / unborn child).

If we also consider that “… physicians currently are the de facto gatekeepers of many wish-fulfilling (…) procedures” [38], the importance of the support that ECs can provide in difficult situations becomes evident. Throughout the analysed cases EC was only requested for very difficult decisions such as performing reproductive treatment for couples with severe chronical illness, terminating healthy pregnancies after IVF (In Vitro Fertilization) or withdrawal of life-sustaining treatment in neonatology. In these situations, an EC can certainly be beneficial to elaborate the decision with the best balance of ethical pros and cons building consensus within the whole team.

## Illustration of three controversial cases

The three cases, which are shown in Table 2, illustrate complex ethical questions arising from parent / patients’ wishes and the reasons why wishes were supported or not [18].

**Table 2:** Three controversial cases

**Case 1 (S6):** EC was demanded during pregnancy by the parents-to-be, who were confronted with the fatal prenatal diagnosis of trisomy 18 in the 25th week of pregnancy. They wished to prepare and ethically justify, with the help of the EC, the postnatal management, i.e. comfort care without life-sustaining measurements for the neonate. They feared that without EC support, their wish might not be respected by clinicians aiming at life protection at all cost possibly using guidelines for justification [39]. **Case 2 (S5, R3):** The head physician requested an EC when a pregnant patient demanded a selective feticide or, if not granted, abortion of her healthy twin pregnancy resulting from fertility treatment, claiming to feel overburdened. Offering selective feticide was denied after EC discussing the conflict between the maternal interests vs. those of the unborn. But the patient underwent abortion in her home country (a “transitional” country with considerable latitude regarding legal abortion). The physician requested another EC when the patient after returning demanded treatment again which upon intense discussion was not offered. **Case 3 (R4)**: An EC was requested by the leading physician when a couple suggested the infertile husband’s father (and thus potential grandfather to the unborn child) should be the sperm donor. Especially the wife, who wished to reach a maximum of genetic similarity to her husband by this approach, strictly rejected any anonymous donor – the usual treatment option. After careful consideration of all options the couple’s wish was by consensus not supported in the EC. However, on further reflection the leading physician changed his decision and allowed the husband’s father to donate his sperm.	

**Case 1:** The couple’s wishes were regarded as well reflected and ethically justifiable. In EC they received support to clarify their ideas and preferred conditions of the delivery convincing the neonatology team of the advance care plan. Retrospectively, the couple evaluated EC and their direct participation in it as most helpful. This case stands out because patient-requested EC with their direct participation are rarely reported [27], esp. in obstetrics [30]; after the experience from this case this practice should be taken into consideration.

**Case 2:** The patient’s request for selective feticide was found to be problematic in light of ethical standards of care, incl. The protection of life, as the termination of the child’s life could not be based on a convincing justification. Moreover, it appeared contradictory to the originally desired pregnancy and instrumentalizing the medical staff for wish-fulfilling medicine. While trying not to moralise the woman’s wish, the team did not concur with serving her persisting ambivalence with repetitive abortions and decided not to offer treatment again. However, it cannot be precluded that the couple obtained the desired service elsewhere.

**Case 3**: Here, the main challenge centred on weighing the couple’s wish for a family-internal sperm donor and, thus, the patient’s autonomy against the potential for conflict, which could threaten the child’s wellbeing. Although the couple’s strong wish to become parents was taken very seriously by all EC participants, the potential threat for the child’s welfare was considered too big. The leading physician, however, agreed to the approach assuming that the family would cope.

## Conclusion

The study shows that treatment decisions in reproductive medicine, obstetrics and neonatology raise substantial ethical questions and disagreements leading to the request for ethics consultation. Clinicians are aware of the duty to respect the patient’s autonomy. However, not every wish for treatment can be supported with ethical reasons. We tried to identify the kinds of wishes of parents (to be) or patients that the clinicians regarded as ethically problematic motivating them to request ethics consultation. Empirical studies on ethics consultation are still rare, especially in this clinical area at the beginning of life. The systematic case series presented here gives insight into the ethical reflection carried out to support the clinicians in their decision-making and counselling. It shows that clinicians, after using ethics consultation, make deliberate decisions that do not “automatically” fulfil the treatment requests of the patients/ parents (to-be).

Despite the clear limitations of our study with its limited number of cases and its retrospective design, we believe it holds valuable observations and highlights that ethics consultation may be helpful at the interphase before and during pregnancy as well as after delivery. Hopefully, we can stimulate other groups to embark on further investigations and more comprehensive studies to create a deeper understanding of this versatile field of work.

## Abbreviations

DNR: Do Not Resuscitate
EC: Ethics Consultations
ICSI: Intracytoplasmic Sperm Injection
IVF: In Vitro Fertilization
